# Establishment of canine mammary gland tumor cell lines harboring PI3K/Akt activation as a therapeutic target

**DOI:** 10.1186/s12917-024-04085-w

**Published:** 2024-05-29

**Authors:** Seo-Young Park, Yeong-Bin Baek, Chan-Ho Lee, Hyun-Jin Kim, Hwang-Phill Kim, Young-Jun Jeon, Jung Eun Song, Su-Bin Jung, Hyo-Jin Kim, Kyeong-Seo Moon, Sang-Ik Park, Chang-Min Lee, Sung-Hak Kim

**Affiliations:** 1 Laboratory of Animal Molecular Biochemistry, Department of Animal Science, Chonnam National University, Gwangju, 61186 Republic of Korea; 2 Laboratory of Veterinary Pathology, College of Veterinary Medicine, Chonnam National university, Gwangju, 61186 Republic of Korea; 3 Laboratory of Veterinary Pathology, College of Veterinary Medicine and BK21 FOUR Program, Chonnam National University, Gwangju, 61186 Republic of Korea; 4 Department of Molecular Medicine & Biopharmaceutical Sciences, Graduate School of Convergence Science and Technology, Seoul National University, Suwon, 16229 Republic of Korea; 5 Department of Integrative Biotechnology, College of Biotechnology and Bioengineering, Sungkyunkwan University, Suwon, 16419 Republic of Korea; 6 Gwangju Animal Medical Center, Gwangju, 62273 Republic of Korea; 7 Laboratory of Veterinary Internal Medicine, College of Veterinary Medicine and BK21 Plus Project Team, Chonnam National University, Gwangju, 61186 Republic of Korea

**Keywords:** Canine mammary gland tumors, Cell lines, PI3K-Akt signaling, EMT

## Abstract

Canine mammary gland tumors (MGT) have a poor prognosis in intact female canines, posing a clinical challenge. This study aimed to establish novel canine mammary cancer cell lines from primary tumors and characterize their cellular and molecular features to find potential therapeutic drugs. The MGT cell lines demonstrated rapid cell proliferation and colony formation in an anchorage-independent manner. Vimentin and α-SMA levels were significantly elevated in MGT cell lines compared to normal canine kidney (MDCK) cells, while CDH1 expression was either significantly lower or not detected at all, based on quantitative real-time PCR (qRT-PCR) analysis. Functional annotation and enrichment analysis revealed that epithelial-mesenchymal transition (EMT) phenotypes and tumor-associated pathways, particularly the PI3K/Akt signaling pathway, were upregulated in MGT cells. BYL719 (Alpelisib), a PI3K inhibitor, was also examined for cytotoxicity on the MGT cell lines. The results show that BYL719 can significantly inhibit the proliferation of MGT cell lines in vitro. Overall, our findings suggest that the MGT cell lines may be valuable for future studies on the development, progression, metastasis, and management of tumors.

**Supplementary Information:**

The online version contains supplementary material available at 10.1186/s12917-024-04085-w.

## Background

Canine mammary gland tumors (MGT) account for almost half of all female canine tumors, contributing to a poor overall survival percentage after surgery [1]. These tumors can be identified by their complicated myoepithelial cell proliferation, some of which lead to the development of complex forms with various mesenchymal components, including cartilage, bone, or bone marrow [2]. Despite sharing many pathological and clinical traits with humans, such as histological grouping, molecular targets, risk factors, clinical progression, and biological behavior, female dogs with MGTs do not respond to therapy in the same way [3, 4]. The most common type of treatment for canine MGTs is surgical resection, but this is not always curative because of the increased risk of local and distant metastases with lymph nodes or vascular invasion. Additionally, since chemotherapy is only based on protocols for treating human breast cancer, it does not significantly increase patient survival [3]. Moreover, there is a lack of information on the molecular markers that can control metastasis and predict how effective anti-tumor drugs will be. Thus, canine cancer cell lines offer an ideal preclinical model for assessing anti-tumor responses, finding cancer-specific signaling pathways, and identifying genes implicated in the development of cancer, such as oncogenes or tumor suppressors.

The nuclear factor kappa B (NF-κB) has a significant impact on inflammation, cell survival, transformation, and cancer development. NF-κB is known to have antiapoptotic, proliferation, motility, and invasion-promoting properties, all of which are essential for normal organ development, including the formation of the mammary gland [5]. According to a study, canine MGT cell lines exhibit activation of the NF-kB signaling pathway. In vitro studies in canine MGT cells, BAY11-7082 (NF-κB inhibitor) significantly reduces the expression of NF-κBα, cyclin D1, and Bcl-2 in a dose- and time-dependent manner. Furthermore, it reduces tumor growth and metastasis, which improves the survival of mouse xenograft models in vivo [4]. On the other hand, a canine MGT cell line, B-CMT, was successfully generated from a canine primary mammary gland tumor and used as a cell model for in vitro drug screening. The B-CMT does not express the human epidermal growth factor receptor 2 (HER2), estrogen receptor (ER), and progesterone receptor (PR). In vitro experiments using cytotoxic drugs, such as imatinib (a tyrosine kinase inhibitor) and rapamycin (a mTOR inhibitor), significantly reduced the proliferation and induced cell cycle arrest at the G1 and G2 phases in B-CMT cell lines [6].

PIK3CA encodes the main catalytic subunit, p110, of the lipid phosphokinase phosphatidylinositol 3-kinase (PI3K) class 1 A [7]. The PI3K family is involved in a variety of biological processes, including cell migration, apoptosis prevention, angiogenesis, and cell proliferation [8]. This pathway is strongly impacted by PTEN loss, PIK3CA, AKT1, and PIK3R1 genetic abnormalities. One of the most common alterations in human cancers is the PIK3CA mutation, which causes persistent activation of p110, enhanced lipid kinase activity, and subsequent activation of Akt [9, 10]. An activated PI3K/AKT/mTOR signaling is linked to cancer cell proliferation, survival, migration, and treatment resistance. Accordingly, PIK3CA mutations (43.1%) and PI3K/Akt pathway abnormalities (61.7%) were frequently seen in canine mammary gland tumors [11]. Interestingly, it has been shown that the PIK3CA mutation is the most common in canine mammary gland tumors [12]. Canine MGTs have unique variant features and molecular characteristics. Thus, it is necessary to establish new, more diverse cell lines that will serve as valuable models for researchers and reflect the features of the original mammary tumor.

This study aimed to establish and describe a new canine MGT cell line in terms of its molecular features, tumorigenicity, and response to cytotoxic drugs. Identification of putative signaling pathways implicated in their development in clinical applications could be employed as targeted treatments and prognostic markers. The MGT cell lines established in this study could serve as an experimental model for studying the biology of canine MGTs.

## Results

### Histological classification of canine MGTs

**Fig. 1:**
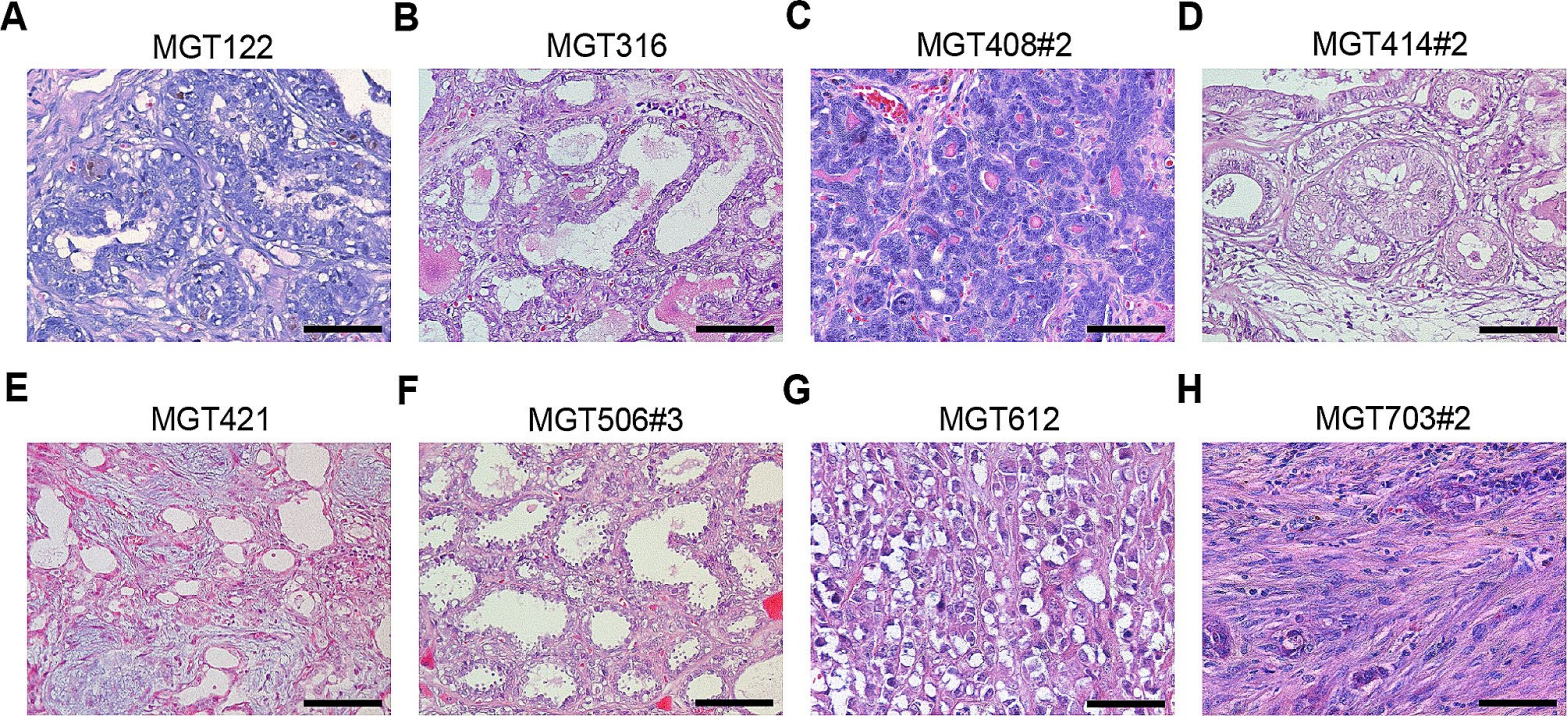
Histological classification of primary canine mammary gland tumors. (**A**-**H**) Tumor paraffin sections, stained with H.E. (Scale bar = 50 μm)

Tissues from canine MGTs were collected from various dog breeds and ages (Table 1). Nuclear and cellular pleomorphism, mitotic index, the presence of a necrosis region, and lymph node metastasis were graded based on H&E staining for histological classification and grade evaluation of tissues (Fig. 1) [13]. Four MGT tissues (MGT122, 414#2, 421, and 612) were classified as complex carcinomas, consisting of neoplastic tubular epithelial cells, and myoepithelial cells, with significant nuclear and cellular pleomorphism. The mitotic patterns observed in these samples varied depending on the malignancy grade. Localized, extensive myoepithelial cell proliferation and epithelial cells with poorly differentiated tubule formations were particularly prevalent in MGT122 tissue (Fig. 1A). Multilobular lesions with tubulopapillary tumor cell patterns were observed in MGT316 (Fig. 1B). The MGT408#2 tissue sample is characterized by in situ carcinoma, which results in the disruption of the normal mammary gland architecture while remaining confined within the basement membrane (Fig. 1C). MGT703#2 exhibits extensive trabecular bone formation at the center of the lesion, with the presence of a few osteoblasts along the trabecular bone spicules (Fig. 1H).

**Table 1:** Information of canine mammary gland tumor tissue sample

Cell line	Age	Breed	Histology	Grade	Score
MGT122	9	Yorkshire	Complex carcinoma	II	6
MGT316	8	Maltese	Tubulopapillary carcinoma	I	4
MGT408#2	8	Maltese	In situ carcinoma	I	3
MGT414#2	11	Poodle	Complex carcinoma	II	7
MGT421	11	Maltese	Complex carcinoma	III	8
MGT506#3	12	Shih Tzu	Tubulopapillary carcinoma	I	3
MGT612	17	Poodle	Complex carcinoma with severe dermal infiltration	II	7
MGT703#2	15	Maltese	Carcinoma and malignant myoepithelioma	III	8

### Primary cell culture from the canine MGTs

**Fig. 2:**
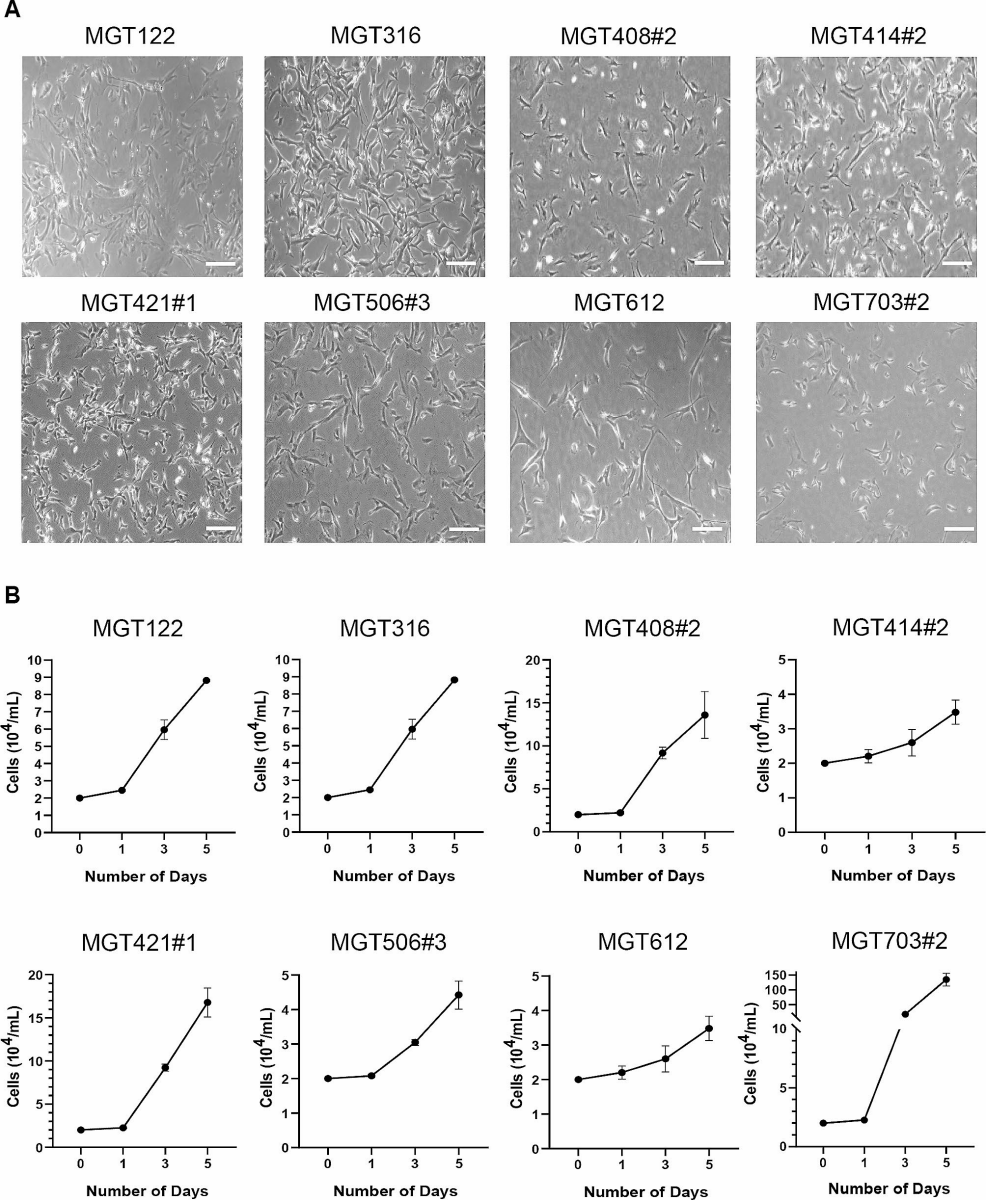
Cell morphology and growth curve of canine mammary gland tumor cell lines. (**A**) Eight cell lines were observed under light microscopy (Scale bar = 100 μm). (**B**) The growth curve of MGT cell lines, the table below shows the doubling time calculated. Each value represents the means of 3 replication experiments

**Fig. 3:**
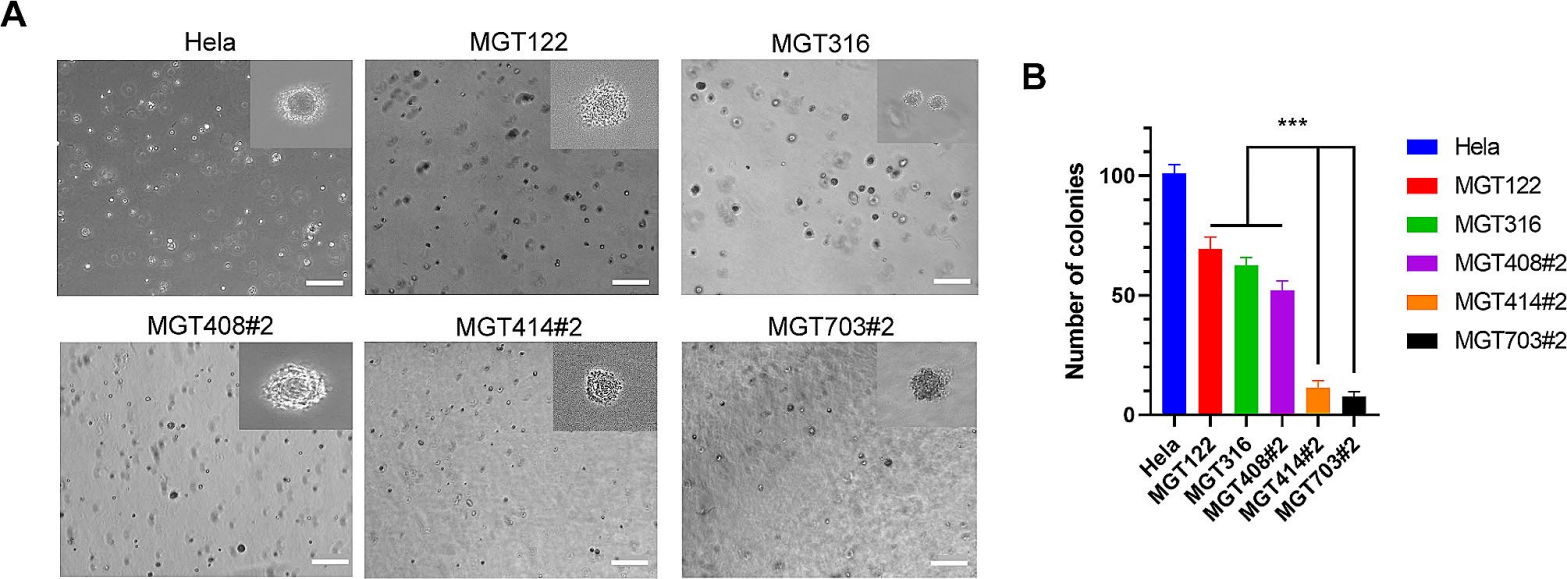
(**A**) Anchorage independent growth ability of canine mammary gland tumor cell lines (Scale bar = 200 μm). The image on the upper right is an enlarged photograph of the cell. (**B**) Quantification of colony numbers. ***P < 0.001 (*n* = 3)

Primary cells were isolated from canine MGT tissues and were maintained in a culture medium containing 10% FBS for several months. The morphology of the 8 MGT cell lines was observed under light microscopy and their population doubling time was determined by a cell counter (Fig. 2A and B). Most of the MGT cell lines retained a fibroblast-like appearance and exhibited varying doubling times ranging from 17 to 135 h. MGT703#2 cells displayed the shortest doubling time at 17.75 h, while MGT414#2 cells showed the slowest doubling time at 151.8 h. The MGT cell lines were assessed using a soft agar assay for their capacity to grow anchorage-independent manner, a quality that cells acquire during tumor development (Fig. 3A). Most of the MGT cell lines used in this investigation have been adapted to grow in soft agar, similar to the human cervical cancer cell line (HeLa). MGT122, MGT316, and MGT408#2 had significantly more colonies as compared to the other MGT cell lines (Fig. 3B).

### Global gene expression analysis in canine MGT cell lines

**Fig. 4:**
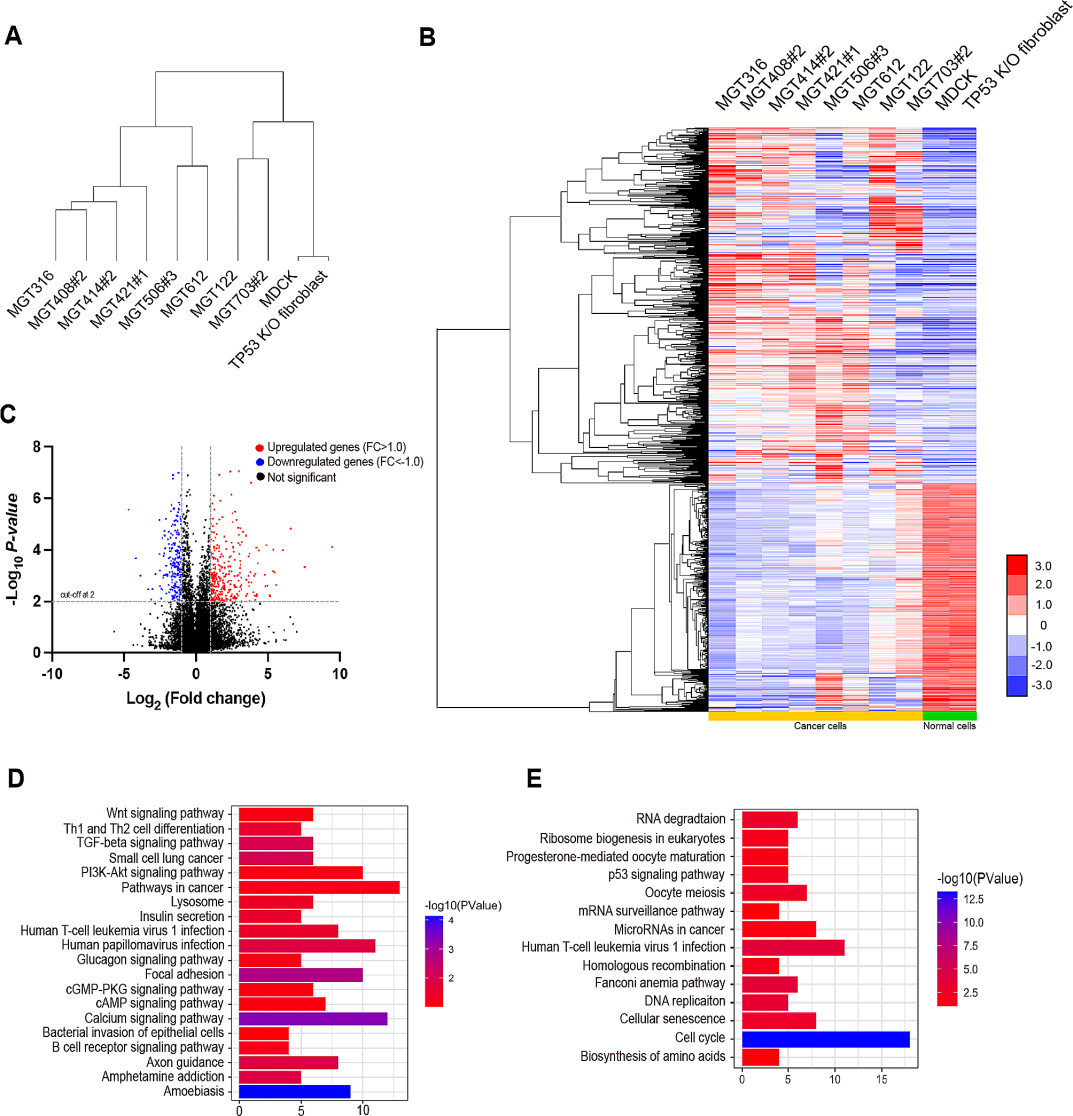
Global gene expression in canine mammary gland tumor cell lines. (**A**) Hierarchical clustering of MGT cell lines, MDCK and TP53 K/O fibroblast. (**B**) Heatmap plot of differentially expressed genes from the comparison of RNA-seq from Canine tumor cells with normal cells filtered by the fold change and p-value (< 0.05). High expression is indicated in red and low expression is indicated in blue. (**C**) Volcano plot of upregulated (*n* = 296), and downregulated (*n* = 212) genes, p-value < 0.05. (**D**) Bar chart for KEGG pathway enrichment analysis of upregulated genes in canine tumor samples. (E) Bar chart for KEGG pathway enrichment analysis of downregulated genes in canine tumor samples

Cluster analysis was used to compare the differential gene expression (DEG) of 10 MGT cell lines to normal samples (MDCK and TP53 K/O fibroblast) using the basal level of all genes identified by RNA-sequencing analysis (Fig. 4A). The majority of MGT cell lines were shown to be more similar to one another in comparison to normal samples based on the dendrogram distances. A heatmap comparing normal groups and MGT cell lines reveals that 296 genes are markedly elevated in MGT cell lines (Fig. 4B). NDUFA4L2, IGFBP2, EPDR1, LOXL3, and FMOD were significantly expressed in MGT cell lines, as demonstrated by the volcano plot (Fig. 4C). According to the Kyoto Encyclopedia of Genes and Genomes (KEGG) analysis, MGT cell lines had lower expression of genes related to p53 signaling pathways, while higher expression associated with TGF-beta, PI3K/Akt, and other cancer-related signaling pathways (Fig. 4D and E). These findings provide insights into the molecular mechanisms underlying the tumorigenesis of MGT cell lines and suggest potential therapeutic targets for canine MGT treatments.

### EMT-related markers expression in canine MGT cell lines

**Fig. 5:**
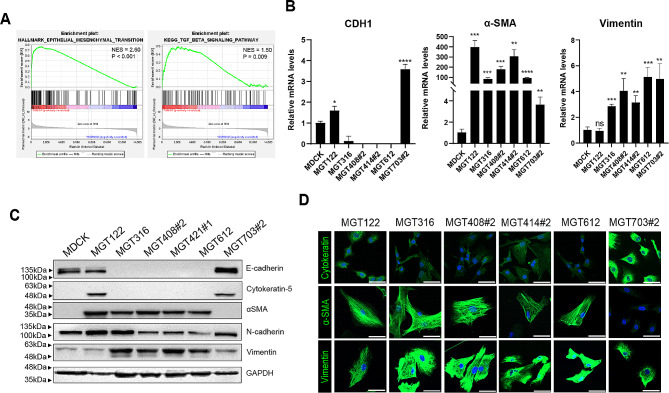
The expression of EMT marker in MGT cell lines. (**A**) Epithelial to mesenchymal transition (EMT), TGF-β signaling pathways were positively correlated in MGT cell lines analyzed by GSEA. (**B**) Quantitative reverse transcription-polymerase chain reaction analysis of CDH1 (E-cadherin1), ACTA2 (α-SMA), VIM (vimentin) in MGT cell lines. ***P < 0.001, **P < 0.01, *P < 0.1 (*n* = 3). (**C**) Western blot images of EMT-related proteins in MGT cell lines. (**D**) Immunofluorescence (IF) staining of epithelial biomarker cytokeratin, EMT biomarkers α-SMA, Vimentin in MGT cell lines. The protein expression of cytokeratin, α-SMA, Vimentin were determined using immunofluorescence stain with IgG-conjugated Alexa Fluor 488 (Green). Nuclei were stained with 4′,6-diamidino-2-phenylindole (DAPI, blue). Scale bar = 50 μm

Gene Set Enrichment Analysis (GSEA) revealed the enrichment of epithelial-to-mesenchymal transition (EMT) and TGF-beta gene signatures in MGT cell lines (Fig. 5A). The qRT-PCR, immunofluorescence assay, and western blot analyses demonstrated the enrichment of EMT-related genes in the MGT cell lines (Fig. 5B, C, D). EMT characteristics were validated using their specific markers such as Cytokeratin, E-Cadherin, α-SMA, and Vimentin. Except for MGT703#2, the majority of the MGT cell lines displayed a low level of cytokeratin and CDH1 but increased expression of vimentin and α-SMA (Fig. 5B, C, D). The result suggested that the established MGT cell lines could be a potential model to study EMT processes during cell invasion.

### PIK3CA mutation (H1047R) status and effects of viability treated potential drug of canine MGT cell lines

**Fig. 6:**
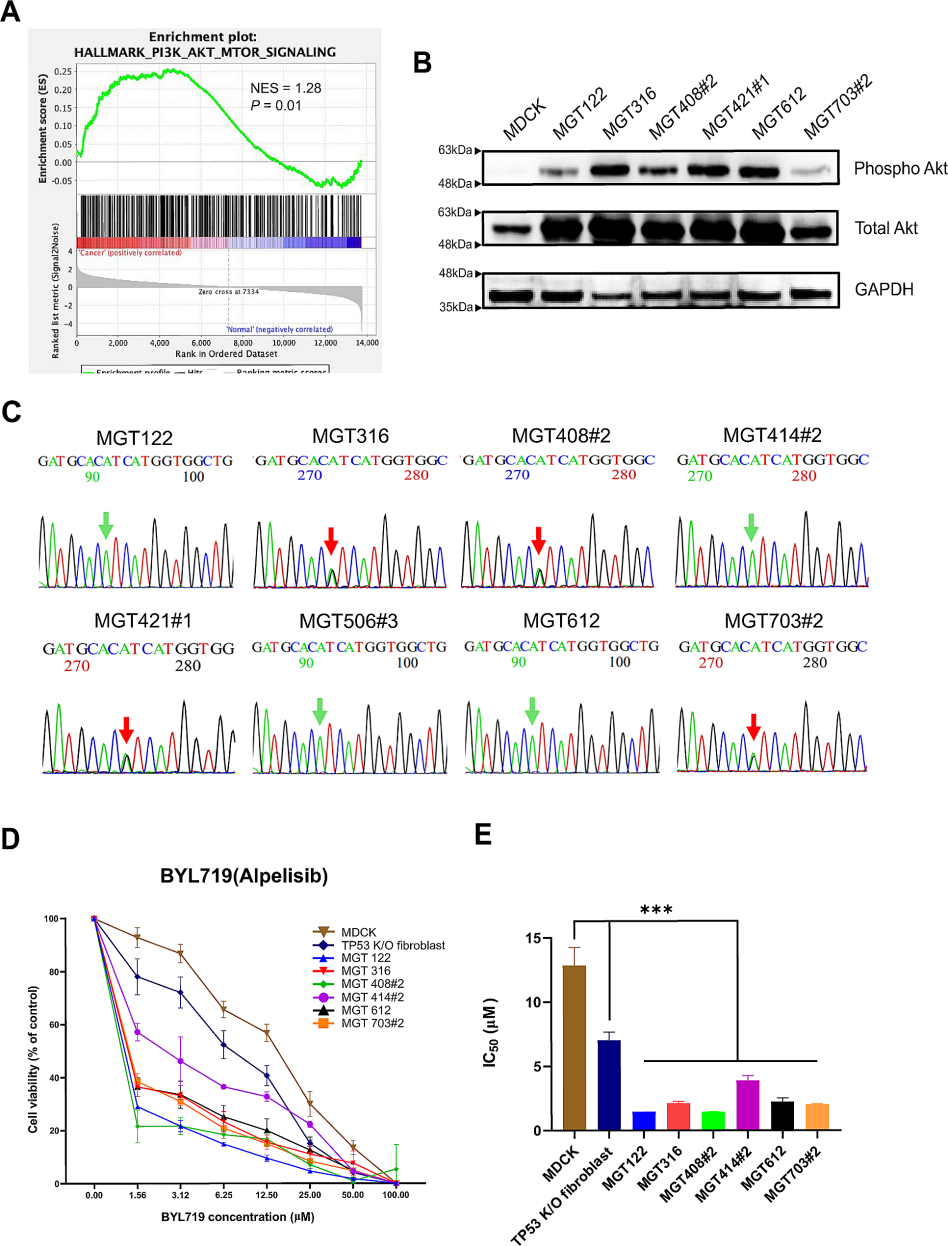
Activation of the PI3K-Akt signaling pathway in MGT cell lines. (**A**) GSEA enrichment plots of MGT cell lines. (**B**) Western blot analysis of phosphorylated-Akt (Ser473), Akt, GAPDH in MGT cell lines. (**C**) Electropherogram of PIK3CA (H1047R) gene mutation status sequencing. (**D**) Antiproliferative activity of BYL719 in MGT cell lines and MDCK, TP53 K/O fibroblast. (**E**) The IC_50_ values of BYL-719 in MGT cell lines and MDCK, TP53 K/O fibroblast. ^***^P < 0.001

GSEA analysis revealed that MGT cell lines have enriched PI3K/Akt gene signatures (Fig. 6A). PI3K/Akt activation was further verified by a western blot using the antibody for phospho-Akt levels (Fig. 6B). One of the frequently occurring mutations in canine mammary tumors is the PIK3CA mutation [12]. We performed DNA sequencing, focusing on the most frequently occurring PIK3CA c.3140 A > G (H1047R) mutation in the MGT cell lines. In MGT316, MGT408#2, MGT421#1, and MGT703#2, the PIK3CA H1047R mutation was verified to be present (Fig. 6C). CCK-8 assay was done to evaluate the correlation between PIK3CA mutation status and BYL719 drug sensitivity in the MGT cell lines. Treatment with BYL719 significantly decreased MGT cell viability in a dose-dependent manner (Fig. 6D). The IC50 values of the negative controls, MDCK and TP53 K/O canine fibroblast, were much higher than those of MGT cell lines, and their viability was reduced after BYL719 treatment (Fig. 6E). Results show no significant difference in the cell viability of wild-type and mutant MGT cells. This suggests that BYL719 can effectively inhibit the proliferation of MGT cell lines regardless of the mutation status.

### In vivo tumorigenesis in mice

**Fig. 7:**
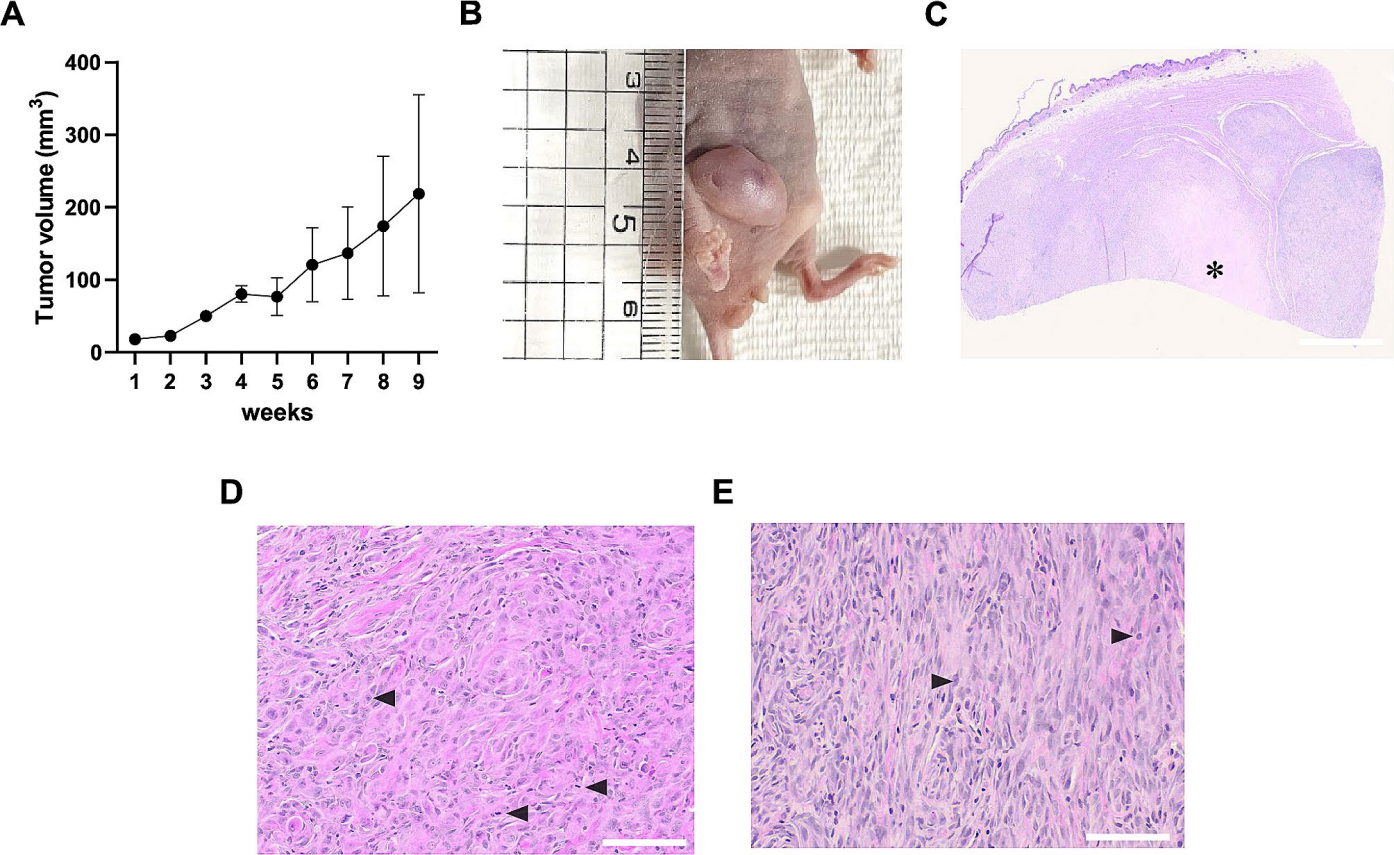
Histopathological features of MGT612 cells through orthotopic injection in Xenograft mouse models. (**A**) Tumor volume of the nude mice. The data are shown as the mean ± SD, *n* = 3. (**B**) Picture of a MGT612 cell xenograft mouse with a tumor in the inguinal fata pad at nine weeks. (**C**) Tumor lesions were multi-lobulated and had severe locally extensive necrosis (asterisk) in the center. (**D**, **E**) The main tumor lesion was characterized by two different cell populations. (**D**) Malignant epithelial cells showed severe loss of tubule formation. (**E**) Spindle-shaped myoepithelial cells were arranged in irregular bundles with a fibrillar matrix. Scale bar = (**C**) 800 μm and (**D**, **E**) 50 μm. Mitotic figures (black arrowhead)

To characterize MGT 612-derived xenograft, 3 × 10^6^ cells were subcutaneously inoculated into BALB/c nude mice at the left inguinal mammary fat pads. The neoplasm was successfully implanted into the orthotopic tissue, gradually increasing size with firm texture over nine weeks (Fig. 7A, B). Next, we performed a histological examination to measure microscopic heterogeneity between the original and implanted tumors. Multi-lobulated tumor lesions had central necrosis observed in the original tumor lesion (Fig. 7C). As expected, the newly formed tumor had two different cell populations, confirming a diagnosis of complex carcinoma (Fig. 7D, E). Overall, these results showed that MGT 612 might create tumors in vivo.

## Discussion

In this study, novel MGT cell lines from canine tumor tissues with various types and grades have been established. These cell lines have fibroblast-like cell morphology and rapid population doubling. RNA-sequencing analysis revealed the expression of gene signatures associated with tumor-specific pathways such as PI3K/Akt and TGF-β. Furthermore, the BYL719 treatment significantly decreased their cell viability, implying that it could be a potential therapeutic drug for the treatment of canine mammary gland tumors.

Canine MGTs represent one of the highly prevalent neoplasms in intact female dogs. Numerous research teams have established cell lines derived from canine mammary tumors to study these lesions comprehensively. Such cell lines derived from canine mammary tumors can be appropriately utilized for various research purposes, including elucidating the etiology of tumorigenesis and screening for anticancer agents. Furthermore, canine mammary tumors exhibit an oncogenic signature remarkably similar to that of human breast cancer, along with notable molecular characteristic resemblances. Given these features, research on canine mammary tumors holds promise for establishing connections with investigations into human breast cancer [11].

The epithelial-mesenchymal transition (EMT) is a biological process in which epithelial cells lose their epithelial features and undergo various metabolic changes to produce a mesenchymal phenotype [14]. Functional annotation and enrichment analysis revealed that MGT cell lines have a high expression of EMT-related gene signatures. It is known that during the EMT process, expression of Vimentin and α-SMA is dramatically increased, while E-Cadherin an epithelial marker is decreased [15]. Furthermore, the MGT cell lines lack E-cadherin expression while expressing high levels of vimentin and α-SMA. Cell-cell adhesions are mediated by E-cadherins, which are calcium-dependent transmembrane glycoproteins [16]. Low E-cadherin levels in canine mammary gland cancers have been linked to higher tumor aggressiveness, metastasis, and short overall survival [17]. The co-expression of E-cadherin and vimentin by MGT122 and MGT703#2 suggests a hybrid EMT state that is beneficial to metastasis and treatment resistance [18]. Hence, the regulatory mechanism needs to be defined or investigated further to fully understand the EMT process.

The PI3K/Akt signaling pathway plays a crucial role in tumor growth, survival, metastasis, and treatment resistance [10]. Constitutive activation of PI3K signaling is known to be a critical step in mediating the transforming potential of oncogenes and tumor suppressors in many tumor types [19]. Activation or inhibition of the PI3K/Akt signaling pathway has been shown to control human cancer cell survival in vitro as well as carcinogenicity, invasion, and metastasis in vivo [20]. Canine mammary gland tumors frequently exhibit aberrant PI3K/Akt signaling activation, according to a recent study [12].

The PIK3CA gene encodes the p110-alpha protein, which is a subunit of the enzyme PI3K. The catalytic subunit that is regulating PI3K is the p110-alpha protein. Certain signaling molecules are phosphorylated by PI3K, which sets off a chain of other events that transmit signals within cells. Numerous cellular processes, such as cell division and proliferation, motility, synthesis of new proteins, intracellular material transport, and cell survival, depend on PI3K signaling. Several cancers, including those of the breast, ovarian, lung, brain, and stomach, have been linked to PIK3CA mutations in this gene [21]. Like human breast cancer, PIK3CA mutations occur frequently in canine mammary tumors [12]. PIK3CA mutations (H1047R) were found in several of our cell lines, and additional research on this is needed.

Since PIK3CA gene alterations are common in breast tumors, they represent rational drug targets [11]. BYL719, a PI3K inhibitor, inhibits p110 roughly 50 times more strongly than other isoforms [22, 23]. BYL719 reduced the viability of MGT cell lines, suggesting that MGT cell lines had abnormal PI3K/Akt signaling. Additionally, BYL719 exerted a significant antitumor effect in canine hemangiosarcoma cell lines carrying PIK3CA mutations. However, no significant impact on canine mammary gland tumor cell lines harboring PIK3CA mutations [5, 24]. In our study, BYL719 can reduce the viability of MGT cell lines but, we did not observe a significant effect on BYL719 based on the PIK3CA mutation status in the MGT cell line. Further mechanistic studies are warranted to elucidate this finding.

Cancer development is marked by anchorage-independent growth, which characterizes the ability of transformed cells to grow without support from a solid surface [25]. MGT cell lines generated colonies on soft agar, showing that their altered phenotype was preserved. To ascertain our MGT cell lines’ capacity for tumorigenesis, we injected our MGT cell lines into the mammary fat pad of BALB/c nude mice. This contributed to improving the translational relevance of our findings to canine MGTs and gave us a more comprehensive understanding of the behavior of our MGT cell lines in vivo. Notably, the MGT612 cell line showed the most rapid tumor formation within 2 months (Fig. 7A, B). Moreover, the transplanted MGT612 cell in mice exhibits histological characteristics that are comparable to those of the original tissue (Fig. 1; Table 1). Histological examination reveals that the tumor lesions exhibited a significant regionally widespread necrosis (asterisk) in the center and were multilobulated (Fig. 7C). These results underscore the tumorigenic potential of MGT612 within the physiologically relevant mammary microenvironment.

However, the other MGT cell lines did not demonstrate tumorigenic capacity in vivo. These results might be similar to the findings of Lainetti et al. and Cordeiro et al. [3, 26]. Successful tumor growth in vivo assay depends on several factors including site injection, cell concentration, the immune system of the animal, or the need for additional hormonal supplementation [27]. Also, although BALB/c nude mice are T lymphocyte deficient, they nevertheless exhibit an immunological response driven by B cells and cytokines like perforins and interferons, which may impede the growth of tumors [28]. Hence, these factors might as well be considered in our future experiments.

On the other hand, regarding the issue of fibroblast cells, we followed the standard protocol for fibroblast contamination elimination [3, 29]. We have provided detailed information on the primary cell culture procedures in the Methods section. Among the generated cell lines, specifically MGT122, culture passages were extended beyond 50. Furthermore, when other cell lines were cultured up to a passage number exceeding 30, they demonstrated consistent utilization in experiments without observable changes in morphology. This indicates their stability and suitability for experimental purposes.

The clonogenicity, gene expression, and drug sensitivity characterization of the canine mammary gland tumor cell lines have shown that they have unique properties. These cells also have a PI3K/Akt signaling signature, implying that the PI3K/Akt pathway is a therapeutic target and a key player in the natural transformation of cancer cells. EMT markers such as Vimentin and α-SMA are also expressed in these cell lines. As such, these MGT cell lines offer a chance to investigate the development of canine mammary gland tumors.

## Conclusion

This study established canine mammary gland tumor cell (MGT) lines from several canine MGT tissues. These cell lines confirmed that the PI3K/Akt signaling pathway was upregulated, and cell viability was reduced by treating BYL719 (Alpelisib) which could inhibit the PI3K/Akt signaling pathway. The findings may provide an experimental basis for investigating the biology, malignancy potential, and therapeutic screening of canine MGTs.

## Methods

### Primary cell culture of canine mammary gland tumors

Canine mammary gland tumors were obtained from the College of Veterinary Medicine at Chonnam National University and local hospitals for primary cell culture. Tumor fragments ~ (1 cm × 1 cm) were dissociated and washed with D-PBS (LB001-02, Welgene, Korea) supplemented with 1% penicillin and streptomycin (P/S, LS202-02, Welgene, South Korea). Gentle MACS Dissociator (130-093-235, Miltenyi Biotec, Germany), enzymatic solution mixed with tissues, was used to dissociate them, and the cells were then rotated for 10 min in CO_2_ incubator at 37 °C, at 5%, utilizing MACs mix Tube Rotation. Then, Cells were separated using a 70 μm mesh filter and then centrifuged at 400 x g for 10 min. The supernatant was removed, and the cell pellet was resuspended and maintained in DMEM/F-12 (Welgene, Seoul, South Korea) or F media (Puricellmania, Seoul, South Korea) supplemented with 10% FBS (Hyclone, UT, USA) and 1% P/S solution. To eliminate fibroblasts, the differential trypsinization method was described in a previous study [3]. HeLa and MDCK cells were purchased from ATCC, and TP53-knockout fibroblasts were kindly provided by Professor H.G. Kim [30]. All cells were incubated at 37 °C with 5% CO_2_. All cells used in the experiment have passage numbers ranging from 10 to 30.

### Cell growth assay

MGT cell lines were seeded in triplicate in a 6-well plate with 2 × 10^4^ cells/well for cell growth assay. Cell counts were performed on days 1, 3, and 5 following the manufacturer’s instructions using an ADAM-MC cell counter (NanoEntek, Seoul, South Korea).

### Soft agar colony formation assay

The bottom layer of a 6-well plate was made up of a 0.6% agar solution in DMEM/F-12 containing 20% FBS, and the top layer was seeded with a single-cell suspension of 5 × 10^4^ cells using a 0.3% agar solution in DMEM/F-12. 2 weeks later, the formation of spheres was observed using CELENA® S digital microscopy (Logos Biosystems, Anyang, Korea). Quantification of colonies involved randomly selecting three distinct areas for monitoring in each well, and individual colonies larger than 20 μm were counted.

### RNA sequencing analysis

RNA was extracted using the RiboEx reagent (GeneAll, Seoul, South Korea). RNA was prepared for the mRNA sequencing library using the MGIEasy RNA Directional Library Prep Kit (MGI) according to the manufacturer’s instructions. The products are then purified and enhanced by PCR to form the final cDNA library. The double-stranded library is measured using the QauntiFluor ONE dsDNA System (Promega). The library is followed by the cleanup of circularization products. The QauntiFluor ssDNA System (Promega) was used to quantify the library. The constructed library was then sequenced on the MGIseq system (MGI). All datasets created and analyzed in this study are deposited in the NCBI’s Gene Expression Omnibus (GSE230271).

### Bioinformatics analysis

Genes with *P* < 0.05 were selected for cluster analysis, complete linkage clustering was carried out using the Cluster 3.0 program, and Java Tree View was used for visualization. Using the GraphPad Prism 8 program (GraphPad Software Inc.), a volcano plot was created to display the differentially expressed genes in tumor samples. GSEA (v4.2.3, available at http://www.broad.mit.edu/gsea/) was used to analyze significant biological pathways.

### H&E staining and histological analysis

Canine MGT tissues were preserved in 10% neutral formalin for 3–4 days at 20 °C. Following sample washing, cutting, and dehydration, an automatic embedding machine was used to perform paraffin embedding. Tissues were sectioned using a microtome with 3 μm thickness and were mounted on slides and then stained with hematoxylin and eosin (H&E). The slides were examined using standard light microscopy. Nuclear and cellular pleomorphism, mitotic index, the presence of a necrosis region, and lymph node metastasis were graded based on H&E staining for histological classification and grade evaluation of tissues [13].

### Cell viability assay

Cells were inoculated with 1 × 10^3^ cells per well in triplicate in 96-well plates and cultured for 24 h until attachment. The cells were then treated with various concentrations of BYL719 (PI3K inhibitor, S2814, Selleckchem, USA). After 72 h, cell viability was measured using the Cell Counting Kit-8 (CCK-8, Dojindo, Japan), following the manufacturer’s instructions. At 450 nm, the fluorescence value was measured using a Synergy HTX multi-mode reader (Biotek, USA). The IC_50_ values were calculated by GraphPad Prism 8 software (GraphPad Software Inc.).

### Immunofluorescence (IF)

An immunofluorescence assay was performed to detect the expression of the α-SMA (1:200, mouse anti-alpha-smooth muscle actin, M0851, Dako, USA), Vimentin (1:200, mouse anti-vimentin, M0725, Dako, USA), Cytokeratin (1:200,mouse anti-cytokeratin, M3515, Dako, USA) in the MGT cell lines. MGT cell lines were seeded in 24-well plates with placed 12 mm coverslips (SPL, Korea). Cells were fixed in 4% paraformaldehyde solution for 15 min at room temperature, permeabilized in 0.2% Triton X-100 (9036-19-5, Sigma-Aldrich) for 10 min, and blocked with 5% bovine serum albumin (BSA) (9048-46-8, Sigma-Aldrich) in D-PBS for 1 h. The cells were incubated with primary antibodies diluted in D-PBS (1:200) with 5% BSA overnight at 4 °C, then incubated with the secondary antibody (1:400) for 2 h at 37℃. Each stage was washed three times for 5 min with D-PBS. All coverslips were mounted with a mounting solution with DAPI (101098-044, Vector laboratories, CA, USA). Slides were imaged with confocal microscopy (Carl Zeiss, Germany).

### Western blot analysis

MGT cell lines were lysed in RIPA buffer (89,900, Thermo Fisher Scientific) supplemented with phosphatase inhibitor cocktail 2 (ApexBio, USA). A bicinchoninic acid (BCA) Protein Assay Kit (23,227, Thermo Fisher Scientific) was used to determine protein content. Proteins were separated on 10% sodium dodecyl sulfate–polyacrylamide gel and then transferred to polyvinylidene difluoride membranes. These membranes were blocked with 5% skim milk in phosphate-buffered saline with Tween®20 (9005-64-5, Sigma) (PBS-T) at room temperature for 1 h and then incubated with corresponding primary antibodies (1:1,000) at 4 °C overnight with moderate shaking. After washing with PBST, membranes were incubated with an appropriate secondary antibody (1:1,000) at room temperature for 1 h. Membranes were then visualized using chemiluminescence (iBright™ 1500, Invitrogen, Thermo Scientific, USA) following the manufacturer’s protocol. Primary antibodies included: Rabbit anti-Vimentin (1:1,000, #5741, Cell signaling, USA), Mouse anti-E-cadherin (1:1,000, #14,472, Cell signaling, USA), Mouse anti-N-cadherin (1:1,000, sc-59,987, Santa Cruz Biotechnology, USA), Mouse anti-Cytokeratin 5 (1:1,000, sc-32,921, Santa Cruz Biotechnology, USA), mouse anti-α-SMA (1:1,000, M0851, Dako, USA), rabbit anti-Akt (1:1,000, #9272, Cell signaling, USA), rabbit anti-p-Akt (1:1,000, #9271, Cell signaling, USA), and mouse anti-GAPDH (1:10,000, #2118, Cell signaling, USA).

### Quantitative reverse transcription-polymerase chain reaction

Total RNA was extracted with RiboEx reagent (GeneAll, South Korea) and purified with a Hybrid-R kit (GeneAll, South Korea) as directed by the manufacturer. A RevertAid First-Strand cDNA Synthesis kit mRNA (Thermo Fisher Scientific, USA) was used to synthesize cDNA from 500 ng of mRNA. SYBR Premix Ex Taq I™ (Takara Bio, Japan) was used for PCR on a CFX96 real-time PCR Detection System (Bio-Rad, South Korea). Results of quantitative reverse transcription-PCR (qRT-PCR) were evaluated as Ct values and quantified using the 2^−ΔΔCt^ method. GAPDH was used as a reference gene to analyze the canine’s gene quantitatively. Primer sequences used for qRT-PCR amplification were as follows: ACTA2 (a-SMA) F: 5’-CATCACCAACTGGGACGACA-3’, R: 5’-GTACATGGCTGGGACGTTGA-3’; VIM (Vimentin) F: 5’-GCGGGAGAAGATGTTGACAATG-3’, R: 5’-CGCAGCCACGCTTTCATATT-3’; GAPDH F: 5’-ATGGTGAAGGTCGGAGTCAA-3’, R: 5’-ATCACCCCATTTGATGTTGG-3’; CDH1 (E-cadherin1) F: 5’-AAATCACATCCTACACCGCC-3’, R: 5’-ATTAACCTCCAGCCAACCG-3’.

### DNA extraction and sequence analysis

Genomic DNA was isolated from cultured cell lines using the G-DEX™ IIc Genomic DNA Extraction kit (iNtRON Biotechnology Inc., 17231, Seoul, Korea), following the manufacturer’s prescribed protocols. To detect the c.3140A > G (H1047R) missense mutation in the coding sequence of canine PIK3CA, the following sequencing primer sets, originated from a previous study [12], were used: F: 5’- CTG GAA TGC CAG AAC TAC AAT C -3′; R: 5’- CTG TTC ATG GAT TGT GCA ATT CC -3′. PCR products were electrophoresed, and specific bands were excised from the agarose gel. Amplified PCR products were extracted from the gel using an EZ-Pure™ Gel Extraction Kit. Ver. 2 (Enzynomics, Daejeon, Korea) DNA samples were submitted to Solgent Inc. (Daejeon, Korea) for sequencing using an ABI PRISM 3730XL Analyzer.

### Mycoplasma detection

Mycoplasma contamination in cell lines was assessed using a LookOut® Mycoplasma PCR Detection Kit (MP0035, Sigma-Aldrich, USA) following the manufacturer’s instructions. Briefly, the supernatant from cultured cells was collected, and a PCR reaction was performed after mixing it with PCR premix and primer mix. PCR products were analyzed by electrophoresis on a 1.2% agarose gel, and the presence of mycoplasma DNA band was visualized under UV light using the Invitrogen™ iBright™ CL750 Imaging System (Thermo Scientific, USA).

### Tumorigenicity assays, tumor growth in immunodeficient mice

For tumorigenicity assays, three 4-week-old female BALB/c nude mice were utilized. Each mouse was inoculated subcutaneously in the left inguinal mammary fat pad with a cell suspension consisting of 3 × 10^6^ MGT cells in 100 µL D-PBS mixed with 100 µL Matrigel. Tumor growth was monitored and measured weekly. After 9 weeks, euthanasia was performed using 1% isoflurane to ensure the mice were unconscious and free of pain prior to sacrifice. The tumor masses were collected and fixed in 4% paraformaldehyde for histological analysis. All procedures involving animals were approved by the Chonnam National University’s Animal Care Committee and complied with the guidelines and regulations set by the Republic of Korea government (CNU IACUC-YB-2023-105).

### Statistical analysis

Microsoft Excel 2013 (Microsoft Inc., Redmond, WA, USA), SPSS 20 (SPSS Inc., Chicago, IL, USA), and GraphPad Prism 8 software (GraphPad Software Inc., La Jolla, CA, USA) were used for statistical analyses. *P-*value < 0.05 indicated statistically significant. The heatmaps were made using Java TreeView (version 1.1.6r4). Significant differences between and among groups were determined by a two-tailed *t*-test and one‐way ANOVA, followed by Tukey’s multiple comparison test.

### Electronic supplementary material

Below is the link to the electronic supplementary material.

Supplementary Material 1

Supplementary Material 2

Supplementary Material 3

## Data Availability

The datasets analyzed during this study are available from the corresponding author upon reasonable request. The datasets generated during the current study are available in the NCBI repository, https://www.ncbi.nlm.nih.gov/sra/, acession numbers SRR28762173- SRR28762180.
