# Supplementary figures and images for: Establishment of canine mammary gland tumor cell lines harboring PI3K/Akt activation as a therapeutic target
Source: BMC Vet Res. 2024 May 29;20:233. doi: 10.1186/s12917-024-04085-w (PMC11134682; doi:10.1186/s12917-024-04085-w)

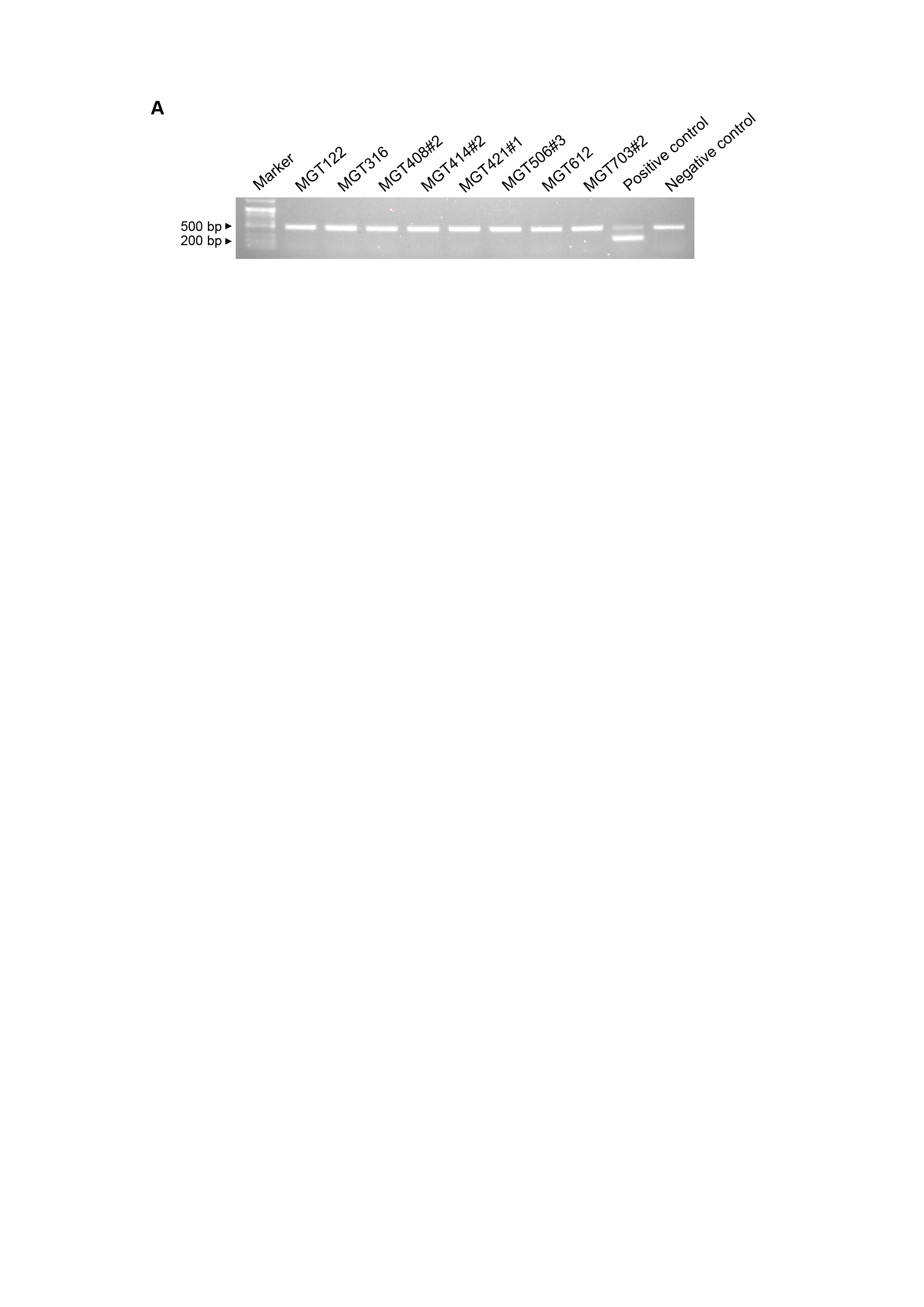

Supplement: Supplementary file 1 — Supplementary Material 1 [file 12917_2024_4085_MOESM1_ESM.tif]
