# Supplementary material for: Establishment of canine mammary gland tumor cell lines harboring PI3K/Akt activation as a therapeutic target
Source: BMC Vet Res. 2024 May 29;20:233. doi: 10.1186/s12917-024-04085-w (PMC11134682; doi:10.1186/s12917-024-04085-w)
Supplement: Supplementary file 2 — Supplementary Material 2 [file 12917_2024_4085_MOESM2_ESM.docx]

**The original blots for the figures**

**Figure 5.**

**
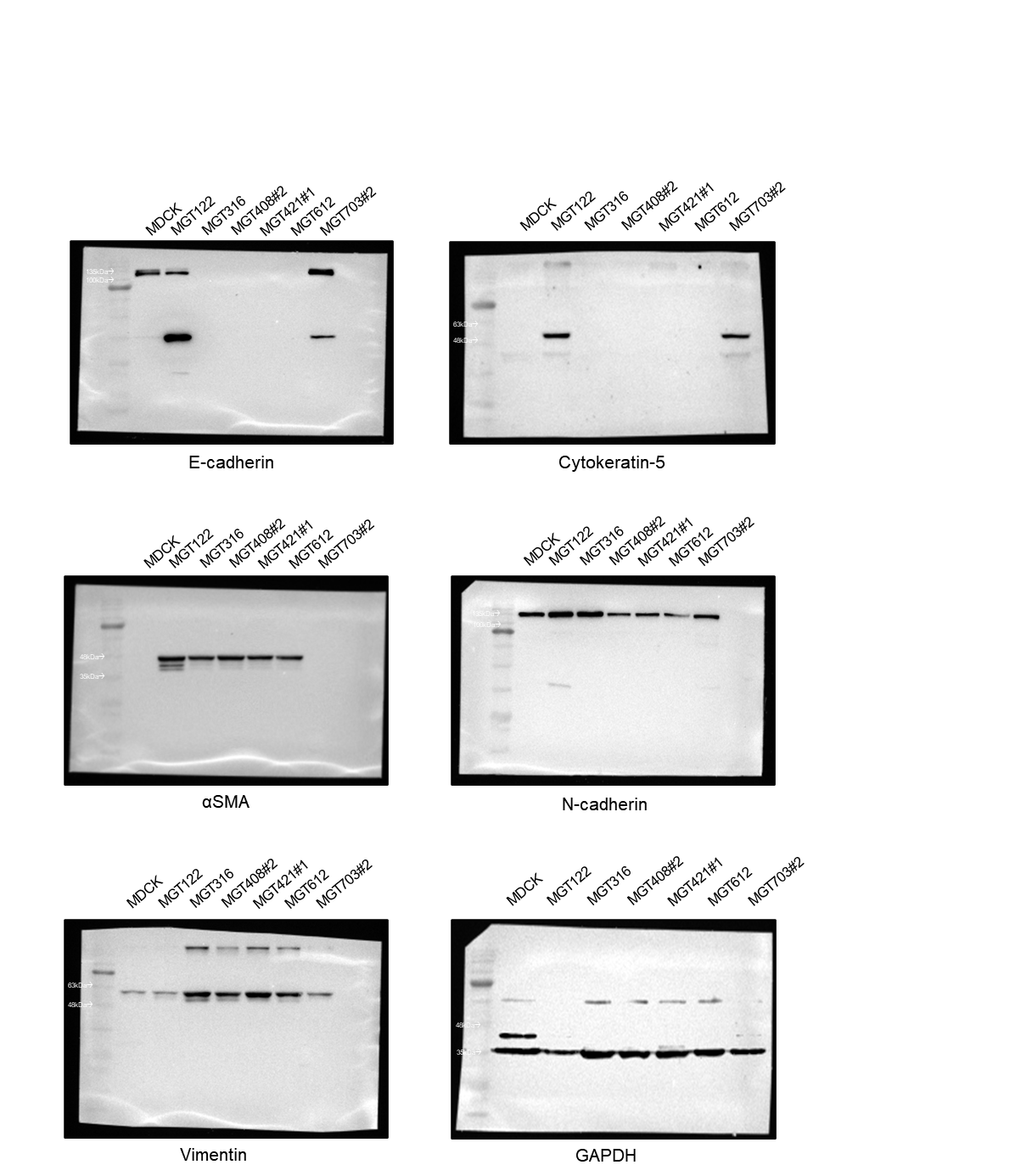
**

Figure 5. Western blot images of EMT-related proteins in MGT cell lines.

**Figure 6.**

MDCK

MGT122

MGT316

MGT408#2

MGT421#1

MGT612

MGT703#2


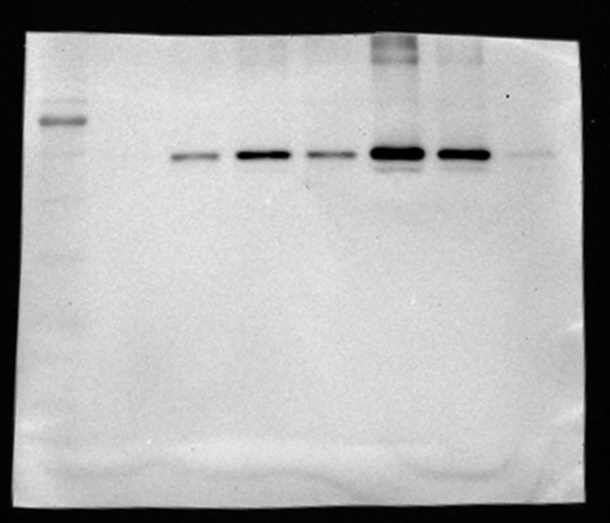


Phospho-Akt (Ser473)

63 kDa

48 kDa


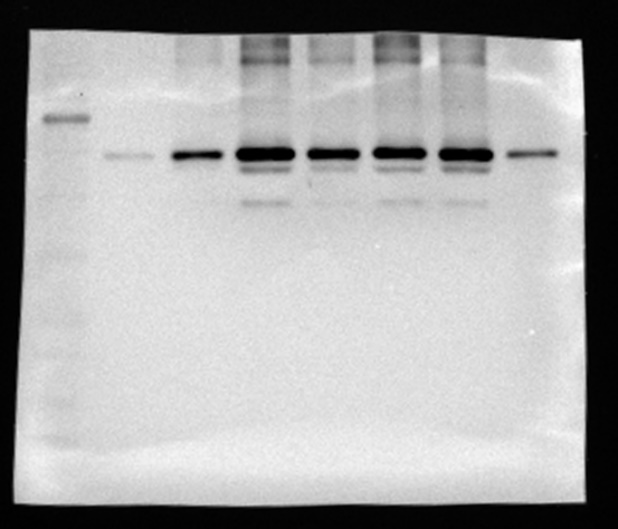


Akt

63 kDa

48 kDa


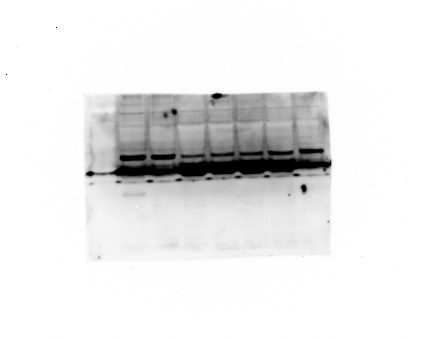


48 kDa

35 kDa

GAPDH

Figure 6. Activation of the PI3K-Akt signaling pathway in MGT cell lines. Western blot analysis of phosphorylated-Akt (Ser473), Akt, GAPDH in MGT cell lines.
