# Supplementary material for: Establishment of canine mammary gland tumor cell lines harboring PI3K/Akt activation as a therapeutic target
Source: BMC Vet Res. 2024 May 29;20:233. doi: 10.1186/s12917-024-04085-w (PMC11134682; doi:10.1186/s12917-024-04085-w)
Supplement: Supplementary file 3 — Supplementary Material 3 [file 12917_2024_4085_MOESM3_ESM.docx]

**Additional Files Legends**

**Additional Files 1: Figure S1.** PCR gel electrophoresis results of MGT cell lines for detection of mycoplasma contamination. (A) No mycoplasma contamination in MGT cell lines.
